# Microbial and Host Metabolites at the Backstage of Fever: Current Knowledge about the Co-Ordinate Action of Receptors and Molecules Underlying Pathophysiology and Clinical Implications

**DOI:** 10.3390/metabo13030461

**Published:** 2023-03-22

**Authors:** Luigi Santacroce, Marica Colella, Ioannis Alexandros Charitos, Marina Di Domenico, Raffaele Palmirotta, Emilio Jirillo

**Affiliations:** 1 Interdisciplinary Department of Medicine, Section of Microbiology and Virology, School of Medicine, University of Bari ‘Aldo Moro’, 70124 Bari, Italy; luigi.santacroce@uniba.it (L.S.); raffaele.palmirotta@uniba.it (R.P.); emilio.jirillo@uniba.it (E.J.); 2 CEDICLO—Interdepartmental Research Center for Pre-Latin, Latin and Oriental Rights and Culture Studies, University of Bari, 70121 Bari, Italy; 3 Department of Precision Medicine, University of Campania ‘Luigi Vanvitelli’, 80138 Naples, Italy; marina.didomenico@unicampania.it

**Keywords:** microbes, microbial antigens, lipopolysaccharide, fever, antipyretic agents, interleukins, prostaglandin E2, thermoregulation

## Abstract

Fever represents an elevation of body temperature, that exerts a protective effect against pathogens. Innate immune cells and neurons are implicated in the regulation of body temperature. Pathogen-associated molecular patterns, i.e., lipopolysaccharides from Gram-negative bacteria and peptidoglycan and lipoteichoic acid from Gram-positive bacteria are exogenous pyrogens, that bind to Toll-like receptors on immune and non-immune cells. The subsequent release of pro-inflammatory cytokines [interleukin-1 (IL-1), IL-6 and Tumor necrosis factor-alpha] and their passage through the brain trigger the febrile response. In fact, neurons of the pre-optic area produce prostaglandin E2 (PGE2), that, in turn, bind to the PGE2 receptors; thus, generating fever. Apart from classical non-steroidal anti-inflammatory drugs, i.e., aspirin and acetaminophen, various botanicals are currently used as antipyretic agents and, therefore, their mechanisms of action will be elucidated.

## 1. Introduction

During infection, several biological systems, even including the immune and the nervous network, become activated to neutralize pathogens. Among symptoms, fever represents a major sign of infection, that is evoked by the stimulation of immune cells via pathogen-associated molecular patterns (PAMPS) [1,2,3]. Immune receptor signaling leads to the production of pro-inflammatory cytokines, that interact with brain endothelial cells, passing to the brain through the vascular organ of lamina terminalis (OVLT) [4]. OVLT is located in the pre-optic area (POA) of the hypothalamus, where thermoregulatory neurons regulate body temperature [5,6]. In this region, the production of prostaglandin E2 (PGE2) and its binding to the PGE2 receptor (EP) generate fever [7,8]. In this respect, there is evidence that loss of EP receptor is followed by a decrease of fever [9,10]. On these bases, the aims of the present review will be to describe major mediators of fever, such as pro-inflammatory cytokines, prostaglandins and oxygen radicals and related receptors [Toll-like receptors (TLRs) and Nucleotide Binding Domain Leucine-Rich Proteins receptors (NLRs)]. Finally, botanicals as antipyretic compounds will be illustrated.

## 2. The Concept of Fever

According to the Commission for Thermal Physiology, the term ‘fever’ consists of the elevation of body temperature above normal ranges (36.5–37.5 °C) [11]. Fever in its origin and its treatment was already an object of study in the ancient world. Fever represents a mechanism of body defense against microbes and microbial products, as well as inanimate substances [12]. In response to antigen penetration, activated immune cells secrete pyrogenic cytokines, which, in turn, induce fever, on the one hand [13]. On the other hand, the temperature sensing neurons in the POA of the anterior hypothalamus trigger an array of responses, such as increased metabolic rate, vasoconstriction, reduced sweating and activation of the immune cells, thus leading to fever [14,15]. Pyrogens are the major agents that are responsible for evoking febrile responses. Among them, endotoxins or lipopolysaccharides (LPS) from the outer membrane of Gram-negative bacteria cell walls are potent inducers of febrile responses, as well as of septic shock, systemic tissue injury and also death in severe cases [16,17]. In this regard, a relevant issue is represented by the administration of biotechnological products and pharmaceuticals contaminated with endotoxins, as well as the implantation of biomedical devices, that may account for the induction of fever [18]. Of note, hyperthermia is an elevation of body temperature, which can reach 41.0 °C, in response to environmental, pharmacologic and endocrine challenges [19]. Contrary to fever, hyperthermia does not respond to antipyretics, since is not provoked by pyretic agents [20].

## 3. Brain Regulation of Fever

**Figure 1:**
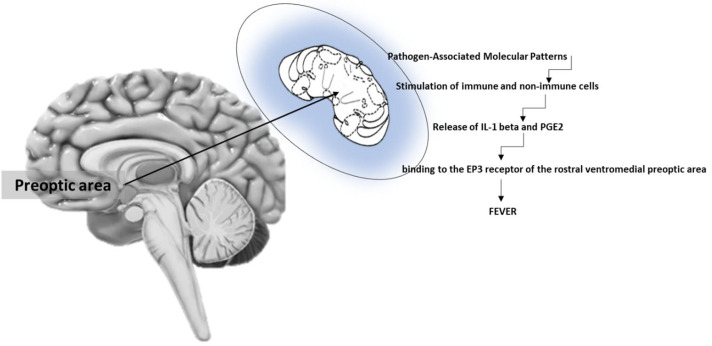
Major mechanisms of fever at the brain level. The preoptic area is endowed with PGE2 receptors (mostly EP3), whose stimulation generates fever.

Pro-inflammatory cytokines [interleukin (IL)-1 beta] lead to the generation of prostaglandin E2 (PGE2), that, in turn stimulates the POA of the hypothalamus [21]. In this respect, evidence has been provided that PGE2 mostly acts at the rostral ventromedial POA (rvmPOA) for fever induction [22]. Three main PGE2 receptors are expressed in the rvmPOA, namely EP1, EP3 and EP4, with EP3 as major players in the generation of fever [9,23]. The intracerebroventricular microinjection of an EP3 agonist also increases body temperature, even if to a lesser extent [24]. Studies on the role of EP1 receptor demonstrated that injection of an EP1 agonist into the rvmPOA induced fever, while pretreatment with an EP1 antagonist hampered the hyperthermic response triggered by PGE2 microinjection into the rvmPOA [25,26]. With special reference to EP4 receptor, the administration of Il-1 beta and LPS up-regulated the EP4 mRNA with expression of Fos protein, as an index of cellular activation in the brain, even including the rvmPOA [24,27]. This may indicate a participation of EP4 receptor in the mechanism of fever. Furthermore, according to recent data [28], newly identified neurons of the rvmPOA are involved in the generation of fever in response to LPS or polyinosinic:polycytidylic acid. The activation of these neurons depends on a paracrine mechanism generated by PGE2, IL-1 beta and CCL2 (also known as MCP-1) produced by ependymal, endothelial, microglial cells and astrocytes. The mechanisms of fever are indicated in Figure 1.

## 4. Receptors of Fever

The so-called PAMPs, such as LPS, lipoarabinomannans, lipoteichoic acid (LTA) and viral DNA, acting as exogenous pyrogens are involved in the mechanisms of fever [29,30,31]. For instance, LPS ca bind the Toll-Like receptor (TLR)-4, which is present on the fenestrated capillaries of the circumventricular organ of the blood brain barrier (BBB) [32]. Once activated, TLR-4 leads to a cascade of events, with cyclooxygenase (COX)-2 production and its conversion into PGE2, that through the BBB stimulates the hypothalamic thermal neurons [33,34,35]. Another set of pathogen-associated recognition receptors is represented by nucleotide-binding domain NLRP1, NLRP3, NLRP7 and NLRC4. These cytosolic sensors detect either pathogens or endogenous danger-associated molecular patterns inside cells, with subsequent formation of multiprotein complexes, called inflammasomes [36]. The NLRP3 inflammasome is activated by an array of stimuli, even including adenosine triphosphate, pore-forming bacterial toxins, particulate structures and lysosome-derived cathepsin B, thus, causing a sequence of events characterized by potassium efflux, reactive oxygen species (ROS) production and decreased levels of cellular cyclic (c) AMP [37]. The NLRP7 inflammasome has been shown to be activated by microbial acylated lipopeptides, exerting a pro-inflammatory role [38]. However, NLRP7 also plays an anti-inflammatory role by repressing NF-kB, as well as sequestering pro-caspase 1 and pro-IL-1-beta [39]. The NLRC4 inflammasome is activated by Gram-negative bacteria, known as type III and IV secretion systems [40]. NLRC4 associates with the neuronal apoptosis inhibitor proteins for activation of caspase-1 and release of IL-1-beta and IL-18 [41]. In this framework, other two NLRs are represented by the protein absent in melanoma 2 (AIM2) and pyrin. AIM2 consists of a N-terminal pyrin domain (PYD) and a C-terminal HIN-200 domain [42]. The binding of double stranded DNA to the HIN-200 domain leads to inflammasome activation. Pyrin is expressed on innate immune cells, such as granulocytes, monocytes and dendritic cells and with its PYD domain binds to the inflammasome adaptor protein, leading to a caspase-1 mediated production of IL-1 beta [43].

## 5. Pyrogenic Cytokines

**Figure 2:**
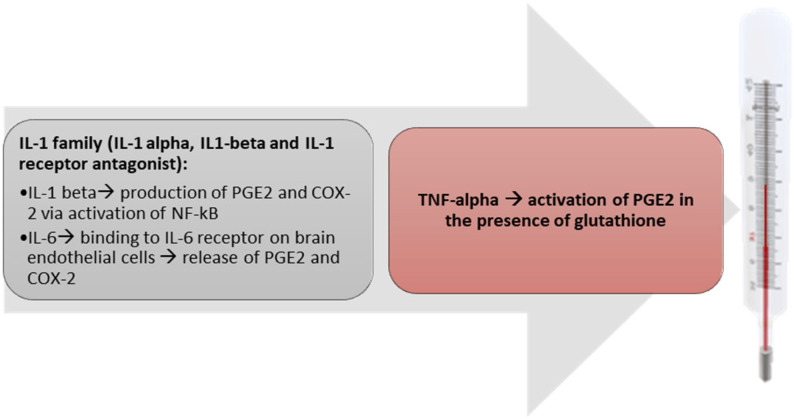
Pyrogenic cytokines. Main mechanisms of fever elicited by IL-1, IL-6 and TNF-alpha are described.

As described in the previous section of this review, the PAMP stimulation of TLRs and NLRs culminates to the release of pyrogenic cytokines, which account for fever induction [44]. Major pyrogenic cytokines encompass IL-1, IL-6 and Tumor Necrosis Factor (TNF)-alpha. With special reference to IL-1, it includes two different molecular forms, IL-1 alpha and IL-1 beta and the IL-1 receptor antagonist (IL-1 RA) [45]. Of note, central or intraperitoneal injection of IL-1 RA was able to suppress LPS-induced fever [46]. The mechanism of IL-1-induced fever relies on its capacity to generate PGE2 and COX-2 production via activation of NF-kB [47,48]. In this respect, IL-1 activates the inhibitor of NF-kB kinase 2, that, in turn, phosphorylates the NF-kB inhibitor alpha with activation of the NF-kB pathway and subsequent production of PGE2 and COX-2 [49]. IL-6 is released by innate immune cells under stimulation by IL-1 or TNF-alpha or activation of TLRs by PAMPs [50]. The pyrogenic activity of IL-6 has been demonstrated through experiments with IL-6 knockout animals or treating animals with IL-6 antiserum, thus, leading to the abrogation of febrile responses [51]. Evidence has been provided that IL-6 binds to IL-6 receptors of brain endothelial cells with production of PGE2 and COX-2 following activation of the STAT3 pathway [52,53]. Quite interestingly, CEACAM1, a molecule expressed on epithelial cells, neutrophils and activated lymphocytes, hampers the early onset of fever thanks to IL-6 macrophage response to LPS [54]. Then, CEACAM1 may represent a valid drug target for antipyretics. TNF-alpha is mainly produced by macrophages in response to LPS, even if T cells and natural killer cells also represent additional sources of this cytokine [55]. Peripheral administration of TNF-alpha to humans and experimental animals causes fever with an increase in PGE2 [56,57]. In the above-cited mechanisms, the presence of glutathione seems to be indispensable since it activates PGE-synthase 1 for production of PGE2 to occur [58,59]. There is evidence that pyrogenic cytokines can skip the BBB, entering the sensory circumventricular organ (CVO) and, then, activating the cytokine receptor of the fenestrated capillaries of the CVO [60]. In turn, vascular and perivascular endothelial cells secrete IL-6 and PGE2. In this framework, it is worth mentioning an alternative theory, namely, the neural transmission of fever. It has been reported that LPS can activate Kupffer cells (KCs), that produce PGE2, stimulating EP3 receptors of the sensory hepatic vagal afferents [61]. Another way of neural transmission of fever relies on the peripheral PGE2 introduction into the nucleus of the solitary tract [62]. In turn, PGE2 is transmitted to POA via the ventral noradrenergic bundle [55]. Norepinephrine contained in the ventral bundle induces the production of PGE2 in the POA via an alpha2-adrenergic receptor-mediated mechanism [61]. Main pyrogenic cytokines are described in Figure 2.

## 6. Mediators of Fever

Studies conducted with LPS demonstrated that PGE2 represents the first initiator of fever. The intravenous injection of LPS is followed by an immediate appearance of PGE2 in inferior vena cava [63]. LPS-activated C5a binds to the C5a receptor 1 on KCs with COX-1-catalyzed PGE2 production [64]. Moreover, brain endothelial cells are an additional source of PGE2 [65]. The phospholipase A2/COX-2/mPGES-1 pathway activates NF-kB and STAT3, that account for PGE2 production, thus, participating in fever maintenance [66]. Both LPS and glutamate induce fever via the increase in oxygen free radicals and ROS [67]. Treatment with hydroxyl radical scavengers or N-methyl-D-aspartate receptor antagonists leads to decrease of oxygen free radicals in OVLT, as well as of fever. Of note, LPS-induced production of ROS activates NF-kB and the expression of COX-2 with elevated production of nitric oxide and PGE2, thus provoking fever [68].

## 7. Fever of Microbial Origin

LPS are major constituents of the outer membrane cell wall of Gram-negative bacteria and their administration mimics the same aspects of Gram-negative bacterial infections. In fact, LPS activate TLR-4, also increasing circulating levels of C-reactive proteins, as observed during Gram-negative infections [69,70]. Following LPS injection, human body temperature rises after 1 h and peaks within 3–4 h [71]. In humans, LPS-induced fever is monophasic, while in rodents two or more phases of fever have been observed [72]. Moreover, in relation to LPS-induced pro-inflammatory cytokine release, substantial differences have been reported between humans and rodents. In humans, intravenous administration of LPS leads to a peak of TNF-alpha 1.5–2 h after the injection, followed by an IL-6 peak 2–3 h after the injection [73,74]. Conversely, in rodents systemically injected with LPS, TNF-alpha peaks after 2 h along with IL-1-beta, with a rise of IL-6 one hour later [75]. The Gram-positive cell wall does not contain LPS and is composed by multi-layers of peptidoglycan (PGN), and teichuronic and teichoic acids [75]. Membrane teichoic acids, hydrophobically anchored to a glycolipid moiety of the plasma membrane, are known as LTAs, which are amphipathic molecules similarly to LPS. PGNs and LTAs signals via TLR-2, that are expressed on monocytes, macrophages and neutrophils [76,77]. LTA also binds to the complement receptor of immunoglobulin superfamily on KCs, which, in turn, binds and captures circulating Gram-positive bacteria, preventing systemic infection [78]. During bacterial infections, PGN and LTA stimulate innate immune cells with the release of pro-inflammatory cytokines, whice promote fever [79]. In a recent report [80], evidence has been provided that the mechanisms of LTA-induced fever are not dissimilar from those observed with LPS, even if LTA effects appear less potent. Viral-derived nucleic acids can interact with TLRs, thus, eliciting febrile responses [81]. In this respect, TLR3 recognizes viral dsRNA, such as reovirus, respiratory syncytial virus, west Nile virus, dengue virus, influenza A virus, Epstein Barr virus and herpes simplex virus [82]. Conversely, TLR7, TLR8 and TLR9 recognize ssRNA in viruses [83,84,85]. Apart from TLRs, the cytoplasmic retinoic acid-inducible gene (RIG)-1, the melanoma differentiation—associated protein (MDA5) and LPG receptors, respectively, recognize different viral ligands. For example, RIG-1 recognizes New-Castle disease virus, Sendai virus, Influenza virus and Japanese encephalitis virus, while MDA5 preferentially recognizes encephalomyocarditis virus and Theiler’ virus [86]. As far as the mechanisms of action of TLR3, TLR7, TLR8, TLR9 and TLR13 is concerned, these endosomal receptors operate either activating the NF-k B pathway or activating type 1 interferon (IFN) signaling via IFN regulatory Factors (IRF) [87,88]. TLR-dependent type 1 IFN-induction follows the production of pro-inflammatory cytokines and depends on mammalian target of rapamycin complex 1 activation [89]. There is evidence that TLR7 activation in dendritic cells is mediated by alpha1beta2 integrins and anabolic conditions which permit type I IFN responses via endosomal positioning [90].

## 8. Fever of Unknown Origin (FUO) and Inflammation of Unknown Origin (IUO)

FUO and IUO are clinical syndromes, which persist up to 3 weeks and more [91]. However, because of the heterogenous nature of FUO and IUO, their precise duration time is still a matter of debate [92]. Moreover, these syndromes are characterized by the absence of an identified cause of fever, despite several investigations, and the persistence of fever for enough time to rule out self-limiting fevers [93]. According to Haidar and Singh, FUO is not a uniform biological event but a common expression of multiple and different clinical conditions, which may vary according to the immune status of the host, patient hospitalization, and travel history [94]. Consequentially, there is no general agreement on time cutoff or diagnostic criteria for FUO. Nevertheless, in order to facilitate the diagnostic approach of fever-related conditions, these syndromes have been divided into four major categories, such as infections, malignancies, non-infectious inflammatory disorders and miscellaneous disorders [95]. This classification, despite its limitations, may be useful to approach the patient with prolonged fever. In general terms, the prevailing perception that inflammatory conditions are the major cause of FUO is contradictory, and, according to two systematic reviews, infections represent the main etiologic factor of FUO [96,97].

Other categories of FUO and IUO are represented by nosocomial fever, fever associated to immunodeficiency and immunosuppressive therapy and FUO in returning travellers [94]. With special reference to immunodeficiency and FUO, millions of people in the United States receive immunosuppressive drugs [monoclonal antibodies, checkpoint inhibitors, and chimeric antigen receptor (CAR) modified T-cells] [98]. In view of biologic variations among immunocompromising conditions, a precise definition of immunodeficiency associated FUO is hard to make. For instance, HIV-associated FUO has been reclassified as FUO in persons receiving antiretroviral therapy (ART) and FUO associated to persons not receiving ART. In fact, ART has converted HIV infection into a chronic disease with very few opportunistic infections, which may account for fever [99]. On the other hand, autoinflammatory and autoimmune disorders, as an expression of an aberrant immune response, represent a frequent cause of FUO [96]. As far the pathogenic mechanisms of action are concerned, autoinflammatory conditions, for example periodic fever syndromes, depend on dysregulated interleukin (IL)-1 beta, and IL-18 responses [100]. Conversely, autoimmune diseases (i.e., autoimmune lymphoproliferative syndrome, rheumatoid arthritis, etc.) are triggered by a type 1 interferon response [101].

In patients with hematological cancers, undiagnosed fever occurs very often because of chemotherapy and bone-marrow transplants. These authors are at risk of prolonged and severe neutropenia, with an absolute neutrophil count of less than 500 per microliter for more than 7 days [102]. In neutropenic patients, fever can depend on translocation of endogenous bacteria and fungi into the blood stream. However, the causative agent of fever is identified in a third of patients in spite of antimicrobial therapy. In the case of neutropenia and fever persistence for more than 7 days, empirical antifungal therapy should be started, with daily examinations, frequent cultures, imaging and nonculture diagnostics. In the absence of neutropenia recovery and prolonged FUO, further treatment with antimicrobial agents should be avoided.

Fever may develop in hematopoietic-cell transplant patients either in the early or in the late post-engraftment period. Under the above-indicated circumstances, the causes of unexplained fever include graft-vs-host disease, opportunistic mold infections, post-transplantation lymphoproliferative disease and cancer relapse.

Quite importantly, fever occurs in 92% of patients receiving CAR T-cell therapy [103]. Usually, fever develops within 3 weeks, and has been ascribed to the cytokine-related syndrome (CKS). Temperatures are very high, and CRS remains a diagnosis of exclusion, when no other causes of fever can be diagnosed early after CART-T cell therapy. In this framework, immune reconstitution syndrome, which occurs on reversal of immune suppression, is a new case of FUO [104]. Such a syndrome represents an aberrant reconstituted immunity to opportunistic pathogens, i.e., cryptococcosis and histoplasmosis. However, in tuberculosis and leprosy, immune reconstitution syndrome can be observed. Furthermore, HIV patients, organ-transplant recipients and persons treated with anti-tumor necrosis factor-alpha are at risk of immune reconstitution-associated FUO.

Despite diagnostic advances, it has been reported that in high-income countries half of the cases of FUO and IUO remain undiagnosed [96,105]. According to a recent study, diagnostic evaluation of FUO and IUO should be based on local disease prevalence, and individual presentations [106].

Furthermore, symptom duration has been considered as a parameter to guide diagnostic testing [107]. Then, this paper clarifies that FUO and IUO patients with symptom duration of more than 12 months often did not receive a final diagnosis.

Erdem and associates [108] conducted a study on FUO in 21 countries with different economic development. These authors based their investigation on the increased life expectancy with higher frequency of elderly people in high economic and social developed countries in comparison with those with limited resources [109,110]. Particularly, age-associated diseases such as hypertension, diabetes and malignancies could be associated to FUO. On these grounds, Erdem and associates [108] selected 788 patients with FUO belonging to low-income, low middle-income, upper-middle income and high-income countries. Results show that FUO causes occur regardless of the economic status of the countries, and this factor does not influence the incidence of FUO worldwide.

As far as management of FUO and IUO is concerned, the first approach is to administer antibiotics and corticosteroids. However, making a precise diagnosis comes first, especially in patients without a rapidly deteriorating clinical status, since they may undergo spontaneous remission [111]. Of note, antimicrobials treatment of patients with FUO and IUO may lead to bacterial resistance, and, in the case of viruses, may result in a false reassurance that the cause of fever has been removed. The same holds true in the case of treatment with corticosteroids, which may lead to fever resolution, but also to a delay of the final diagnosis.

## 9. Antipyretic Molecules

Glucocorticoids are hormones released by the adrenal cortex, which are endowed with anti-inflammatory function, mostly inhibiting the release of pro-inflammatory cytokines [112]. In rats administered with LPS, an increase in corticosteroids was detected for the control of endotoxin-mediated fever [113]. In addition, evidence has been provided that corticosterone reduces fever, inhibiting the release of COX2 and PGE2 from the POA of hypothalamus [114]. Similarly, the corticosterone inhibited LPS-induced activation of NF-kB and the related production of IL-6.

Ghrelin is a 28 amino acids peptide, that is secreted by gastric oxyntic mucosa and inhibits LPS-mediated fever. Such an inhibitory mechanism relies on the ability of ghrelin to decrease the production of PGE2, IL-1 beta, TNF-alpha and IL-6, while increasing the release of the anti-inflammatory cytokine IL-10 in response to LPS [115,116,117]. It has been reported that the ghrelin inhibitory function relies on the prevention of translocation of p65 subunit of NF-kB for pro-inflammatory cytokine translocation to occur.

Melatonin, a secretory product of the pineal gland, has been shown to reduce the expression of major pro-inflammatory and pyrogenic cytokines in response to LPS [118]. The anti-inflammatory activity exerted by melatonin has been evaluated using neuronal stem cells (NSCs) treated with LPS [119]. In this test system, melatonin increased the survival of NSCs, reducing NO-mediated damage. Alpha-melanocyte stimulating hormone (MSH) is an endogenous melanocortin, that is endowed with anti-pyretic effects [120]. Alpha-MSH antipyretic activity leads to the inhibition of leukocytic pyrogens without antagonizing PGE2 or arachidonate hyperthermia [121]. Moreover, its activity depends on central melanocortin receptors with no involvement of the pituitary-adrenal axis [122].

Vasopressin is a neurohormone secreted by the paraventricular nucleus in the hypothalamus. The antipyretic effect of vasopressin has been detected, using RAW264.7-treated with LPS [123,124]. In this test system, addition of arginine vasopressin inhibited activation of the NF-kB pathway and release of pro-inflammatory cytokines, as well as of PGE2.

Among non-steroidal anti-inflammatory drugs, aspirin is the most well-known antipyretic compound also endowed with anti-inflammatory and anti-platelet activities. Aspirin effects depend on the reduction of PGE2 and, particularly, a novel nitric oxide and hydrogen sulfide-releasing hybrid has been demonstrated to be a safer alternative to aspirin in terms of diminished lipid peroxidation and gastric damage [125]. Furthermore, acetaminophen, another antipyretic drug, acts through inhibition of COX2 and PGE2 either in vitro or in vivo [126]. Nowadays, botanicals have gained more attention for their antipyretical activity. Particularly, research has been focused on their mechanisms of action. Curcumin, as a major component of *Curcuma longa* roots, has been shown to exert anti-inflammatory effects by suppressing TLR4 and down-regulating the NF-kB pathway with reduced transcription of pro-inflammatory cytokines [127,128,129,130,131]. In a recent paper, evidence has been provided that curcumin pretreatment mitigates LPS-induced fever in rodents through elevation of the nuclear factor-erythroid 2 related factor (Nrfr) [132]. Actually, Nrfr in response to LPS translocates to the nucleus, regulating the expression of antioxidant and anti-inflammatory genes [133].

**Figure 3:**
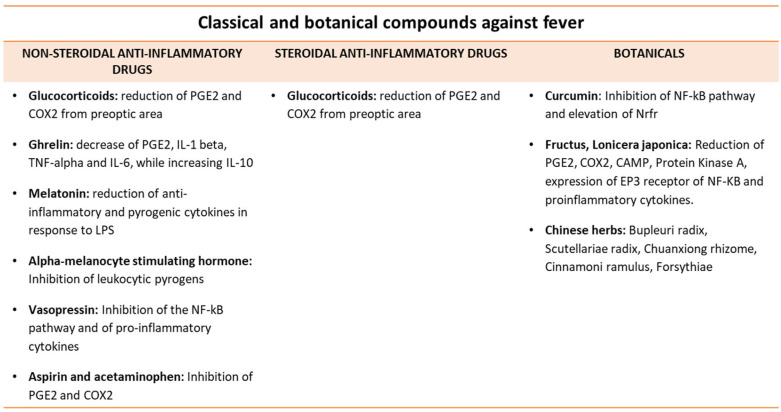
Major antipyretic compounds. Classical compounds and botanical effects on fever are described.

Chinese herbal medicines [Bupleuri radix (Br), Scutellariae radix (Sr), Chuanxiong rhizome (Cx), Cinnamoni Ramulus (Cr), Forsythiae fructus (Ff) and Lonicera japonica flos (Lj)] have been investigated for their antipyretic activity. Br contains several compounds, such as essential oils, triterpenoid saponins, flavonoids, lignans, fatty acids and sterols [134,135,136]. In one study, it has been reported that the antipyretic effect of Br resides in its ability to decrease cAMP in the cerebrospinal fluid [137]. In another study, intracerebroventricular injection of saikosaponin (SSA) could exert antipyretic activity, reducing protein kinase A and cAMP in the hypothalamus [138]. Furthermore, SSA could hamper the NF-kB pathway, suppressing the phosphorylation of inhibitory IKBA and expression of pro-inflammatory cytokines [139]. In the case of Sr, baicalin (a flavonoid) represents the main component that reduces fever [140]. Baicalin inhibits the N-methyl-D-aspartate receptor-dependent hydroxyl radical pathways in the hypothalamus with abrogation of TNF-alpha dependent fever in response to LPS [141]. Furthermore, baicalin inhibits the expression of COX-2 in RAW 264.7 macrophages and then, the production of PGE2 [142]. With special reference to Cx, its essential oils play an antipyretic activity either inhibiting the expression of COX2 and the production of PGE2 in the rat hypothalamus or suppressing the TNF-alpha-mediated NF-kB activation [143,144]. Cr exerts its antipyretic activity via cinnamaldehyde, which inhibits yeast-induced fever in rats, reducing the PGE2 in the hypothalamus or hampering the IL-1-mediated release of PGE2 [145]. Ff also possesses an antipyretic effect owing to its content in essential oil and forsythoside (an inhibitor of pro-inflammatory cytokines)) [146]. Forsythoside can down-regulate cAMP and PGE2 levels in the hypothalamus with the reduction of TNF-alpha, blocking the LPS/TLR4 signaling pathway [147]. Lj is enriched in essential oils, iridoids, flavones, saponins and organic acid. In rabbits with IL-1 beta-mediated fever, Lj exerts antipyretic activity, inhibiting the expression of the receptor EP3 in pre-optic anterior hypothalamus [148]. Finally, for its content in bifido-flavonoids, LJ inhibits the NF-kB pathway with abrogation of COX2 and PGE2 synthesis [149]. The main antipyretic compounds are indicated in Figure 3.

## 10. Future Perspectives

Among exogenous pyrogens, LPS represent potent riggers of febrile responses and those derived from the gut are the most abundant aliquot [150]. In this respect, subversion of oral and intestinal microbiota facilitates the passage of LPS through the BBB, ultimately, reaching the brain [151,152]. This mechanism may account for some cases of fever of unknown origin [153]. Botanicals are good alternatives for NSAIDs for treating fever. Currently, polyphenols and Chinese herbs are used as anti-inflammatory and antipyretic agents in patients with mild symptoms of COVID-19 [154,155].

## 11. Conclusions

Conclusively, fever is a defense mechanism against pathogen invasion. The neuroimmune networks participates in the mechanisms of fever with peripheral and central cytokines acting upon thermoregulatory brain neurons. However, the diagnosis of FUO and IUO is very hard, but a minimal workup should be done before a patient is considered to have FUO and IUO. Furthermore, it should be kept in mind that specific testing performed may vary based on several factors, even including epidemiologic, host and resource-related factors. Therefore, the selection of compounds with the above listed properties may represent novel therapeutic options as adjuvant treatment for several infectious diseases, including COVID-19 patients.

## Data Availability

Data is available within the article.

## Abbreviations

BBB	Blood Brain Barrier
cAMP	Cyclic AMP
COX-2	Cycloxygenase 2
CVO	Circumventricular Organ
EP	PGE2 receptor
IFN	Interferon
IL	Interleukin
IRF	IFN Regulatory Factors
IL-1RA	IL-1 Receptor Antagonist
LPS	Lipopolysaccharide
LTA	Lipoteichoic Acid
MDA5	Melanoma Differentiation-Associated Protein 5
MSH	Melanocyte Stimulating Hormone
NLR	Nucleotide Binding Domain Leucine-Rich Protein Receptor
NO	Nitric Oxide
Nrfr	Nuclear Factor- Erythroid 2 Related Factor
NSCs	Neuronal Stem Cells
PAMPs	Pathogen-Associated Molecular Patterns
PGE2	Prostaglandin E2
PGN	Peptidoglycan
POA	Preoptic area
PYD	Pyrin Domain
OVLT	Organ Vascular Lamina Terminalis
RIG-1	Retinoic Acid-Inducible Gene-1
ROS	Reactive Oxygen Species
rvmPOA	Rostral Ventromedial POA
SSA	Saikosaponin
TLRs	Toll-Like Receptors
TNF	Tumor Necrosis Factor
